# Xianyu capsule ameliorates neuroinflammatory and glycerophospholipid metabolism in lithium-pilocarpine-induced acute epilepsy

**DOI:** 10.3389/fnut.2025.1625533

**Published:** 2025-08-13

**Authors:** Dongsheng Yu, Shuang Li, Xiaoping Li, Xiaodan Zhang, Danfeng Guo

**Affiliations:** ^1^Department of Chinese Medicine, The First Affiliated Hospital of Zhengzhou University, Zhengzhou, Henan, China; ^2^Department of Neurology, The First Affiliated Hospital of Zhengzhou University, Zhengzhou, Henan, China; ^3^Department of Hepatobiliary and Pancreatic Surgery, The First Affiliated Hospital of Zhengzhou University, Zhengzhou, Henan, China; ^4^Henan Key Laboratory for Digestive Organ Transplantation, The First Affiliated Hospital of Zhengzhou University, Zhengzhou, Henan, China

**Keywords:** Xianyu capsule, epilepsy, neuroinflammation, glycerophospholipid metabolism, gut microbiota

## Abstract

**Objective:**

Xianyu capsule (XYC) is a commonly used traditional Chinese medicine in the clinical treatment of epilepsy, with significant curative effect and good safety. However, its mechanism of action remains poorly understood. This research employed a multi-omics approach to systematically evaluate the anti-epileptic efficacy of XYC and elucidate its underlying mechanisms.

**Methods:**

Epilepsy rat model was established by lithium-pilocarpine hydrochloride injection. XYC was administered and the effects and mechanism was analyzed with H&E and Nissl staining, TUNEL assay, ELISA assay for inflammatory cytokines, 16S rDNA, non-targeted metabolomics and network pharmacology. The potential target were experimentally validated with RT-qPCR and Western blotting analysis.

**Results:**

XYC administration ameliorated the pathological changes and neurons apoptosis of brain hippocampus CA1 region, with reduced MDA and increased SOD and CAT levels in hippocampus, and decreased inflammation cytokine in serum. 16S rDNA sequencing revealed distinct gut microbial restructuring in XYC-treated epileptic models, characterized by phylum-level alterations in lipid-associated taxa (*Tenericutes*, *Patescibacteria*, *Epsilonbacteraeota*, *Proteobacteria*) and genus-level modulations (*Lactobacillus*, *Ramboutsia*, *Staphylococcus*). Serum metabolomics identified 149 differentially expressed metabolites positively correlated with XYC’s anti-epileptic effects, predominantly enriched in glycerophospholipid metabolic pathways. Network pharmacology identified AKT1, INS, and IL-6 as pivotal mediators of XYC’s therapeutic effects, which were subsequently validated with Western blotting and ELISA assay.

**Conclusion:**

Our results proved that XYC exerted favorable effect on epilepsy by modulating the gut microbiota and serum lipid metabolic, especially neuroinflammation and glycerophospholipid metabolism by regulating the AKT1, INS and IL-6 expression levels. In addition, targeting neuroinflammatory pathways and modulating glycerophospholipid metabolism may represent a promising therapeutic strategy for epilepsy management.

## 1 Introduction

As a disabling neurological condition affecting people of all ages, races, and geographical regions, epilepsy manifests in over 65 million patients worldwide, with China accounting for 15.4% of the total burden (10 million cases). The hallmark neuropathological feature involves paroxysmal cerebral network dysregulation caused by pathological neuronal hypersynchrony. With an annual incidence of approximately 650,000 new cases worldwide, this condition demonstrates significant etiological heterogeneity and substantial heritability (1–3). Epilepsy is a disease associated with various etiologies and risk factors, and exhibits high heritability‌. Clinical studies indicate that, nearly 30–40% epilepsy cases demonstrate resistance to conventional antiepileptic drugs‌, while 60% of epileptic seizures are idiopathic (4). While current clinical arsenals comprise more than 30 antiseizure drugs, 30–35% of epilepsy patients develop pharmacoresistance, with seizure recurrence rates exceeding 50% despite optimized antiseizure drugs regimens (5). Therefore, it is necessary to further explore the pathogenesis and effective treatment methods of epilepsy.

Accumulating evidence indicates that seizure episodes are closely associated with a cascade of inflammatory cytokine upregulation, which drives neuroinflammatory processes, exacerbates cerebral pathophysiology, and facilitates ictal propagation. Notably, immune responses have been implicated in initiating sustained neuroinflammatory cascades that contribute to the molecular mechanisms underlying epileptogenesis (6, 7). And there is a potential interaction between gut microbiota and neuroinflammation in central nervous system (CNS) disorder diseases, including Alzheimer’s disease, multiple sclerosis and Parkinson’s disease (8, 9). The gut microbiota modulates CNS activity via microbial metabolites, neurotransmitters, and immune-inflammatory signaling, while the CNS influences gut microbial composition through neuroendocrine pathways, conversely. This bidirectional communication system is termed the gut-brain axis (10). The bidirectional gut-brain axis communication is significantly mediated by intestinal flora metabolites, with short-chain fatty acids (SCFAs), lipopolysaccharides (LPS), and amino acids serving as critical mediators in modulating neuroinflammation and maintaining CNS homeostasis. These microbiota-generated metabolites modulate critical neural mechanisms including blood–brain barrier integrity maintenance, myelin formation, neuronal regeneration, and microglial development. Furthermore, they modulate various aspects of animal behavior, demonstrating the microbiota-brain axis’s critical role in CNS functionality (11–13). The gut-brain axis functions bidirectionally, with the CNS modulating gut physiology through autonomic pathways. Neural control of digestive processes—including secretion of intestinal peptides and mucus—along with CNS-mediated regulation of mucosal immunity, collectively shapes the gut microenvironment. These neurogenic influences create dynamic habitat conditions that directly affect microbial colonization and population dynamics (14, 15). Epileptic patients, particularly pharmacoresistant cases, demonstrate characteristic gut microbial dysbiosis and topographical reorganization relative to healthy controls (16, 17). These dysbiotic patterns may reflect disease-specific microbial signatures associated with seizure pathogenesis.

Recent studies have revealed that both individuals with epilepsy and preclinical animal models exhibit characteristic gut dysbiosis. Liu et al. (18) demonstrated that epilepsy patients exhibit microbiome dysbiosis, while the clinical findings were corroborated in animal models, where metagenomic analysis of epileptic rats showed an elevated *Bacteroidota*-to-*Firmicutes* ratio compared to controls (19). Complementing these results, Oliveira et al. (20) reported reduced microbial diversity (lower Chao1 index) in lithium-pilocarpine-induced epileptic rats, along with increased *Desulfobacterota* and decreased *Patescibacteria* abundance at the phylum level. The consistent dysbiosis patterns across species suggest intestinal microbiome emerges as a dual-function modulator in epilepsy pathogenesis (17, 21). Notably, fecal microbiota transplantation has demonstrated anti-seizure efficacy in human patients, canines, and rodent models (22, 23), collectively validating the microbiota-brain axis dysregulation hypothesis in epileptogenesis.

Disordered lipid homeostasis constitutes a central etiological factor in epileptogenesis, manifesting specifically through dysregulated metabolism of triglycerides (TGs), cholesterol (CHOL), and fatty acid (FA) (24). Clinical and preclinical studies demonstrate that epilepsy induces lipid metabolic reprogramming through FA-associated signaling pathways in both pediatric patients and rodent models (25, 26). This metabolic disruption manifests as elevated TG accumulation, impaired CHOL homeostasis (inhibited efflux and enhanced influx), and reduced FA *β*-oxidation. These alterations promote reactive oxygen species (ROS) generation, exacerbating neuroinflammation and neuronal apoptosis (27). Additionally, epilepsy progression involves abnormal metabolism of SLs (crucial for neurodevelopment) and phospholipids (essential membrane components) (28, 29). The ketogenic diet (KD) has emerged as an effective therapeutic strategy for epileptic pharmacoresistant cases. This high-fat, adequate-protein, low-carbohydrate intervention induces ketone bodies (*β*-hydroxybutyrate, acetoacetate, and acetone) concomitant with elevated serum CHOL and TG concentrations. The KD demonstrates anticonvulsant efficacy via multimodal mechanisms: stabilizing neuronal hyperexcitability, optimizing mitochondrial energetics, and restructuring enteric microbial ecology (30, 31). These findings position lipid metabolism modulation as a promising therapeutic paradigm for epilepsy management (32).

Xianyu Capsule (XYC), a traditional Chinese medicine (TCM) formulation, is clinically employed as an antiepileptic therapeutic agent, formulated by modifying and combining two classical TCM prescriptions: Qianzheng San (Symmetry-Restoring Powder) and Tianma Gouteng Yin (Gastrodia-Uncaria Decoction), with phlegm-resolving and consciousness-restoring, sedative and anticonvulsant, wind-extinguishing and spasmolytic effect. XYC containing 16 medicinal compounds (*Hedysarum Multijugum Maxim., Codonopsis Radix, Radix Salviae, Radix Bupleuri, Ziziphi Spinosae Semen, Polygala tenuifolia Willd., Rhizom Gastrodiae, Uncariae Ramulus Cumuncis, Curcumae Radix, Arisaema Cum Bile, Angelicae Sinensis Radix, Acoritataninowii Rhizoma, Bombyx Batryticatus, Massa Medicata Fermentata, licorice and Typhonii Rhizoma, and Glycyrrhiza uralensis Fisch.*). XYC has been used in the treatment of epilepsy for more than twenty years in clinical settings (SFDA approval number: Z20025728). However, the precise molecular underpinnings remain incompletely elucidated.

In this study, we employed a lithium-pilocarpine hydrochloride induced rat model of epilepsy to evaluate the neuroprotective effects of XYC. A multi-omics approach was implemented, combining untargeted serum metabolomics, gut microbiomics, and network pharmacology analysis to comprehensively assess XYC’s protective mechanisms.

## 2 Materials and methods

### 2.1 Chemical and reagents

Xianyu Capsule (XYC, Lot #20220401) was obtained from Xi’an Chiho Pharmaceutical Co., Ltd. (Xi’an, China). Pilocarpine hydrochloride (#HY-B0726) was purchased from MCE (Shanghai, China). Malondialdehyde (MDA, #A003-1) and Superoxide dismutase (SOD, #A001-3) assay kits were purchased from Nanjing Jiancheng Bioengineering Institute (Nanjing, China). TUNEL BrightRed Apoptosis Detection Kit (#A113-02) were obtained from Vazyme Biotech Co., Ltd. (Nanjing, China). Akt1-Specific Recombinant antibody (#80816-1-RR) and Alpha Tubulin polyclonal antibody (#11224-1-AP) were acquired from Proteintech (Wuhan, China). ELISA assay kit for IL-6 (#E-EL-R0015), TNF-*α* (#E-EL-R2856), IL-1β (#E-EL-R0012) were obtained from Elabscience Biotechnology Co., Ltd. (Wuhan, China).

### 2.2 UPLC-MS analysis of XYC

XYC constituents were analyzed with UPLC-Orbitrap-MS as previously described (33). Chromatography on Waters HSS T3 column (100 × 2.1 mm, 1.8 μm). High-resolution mass spectrometric data were acquired using Q Exactive HFX Hybrid Quadrupole-Orbitrap instrument (Thermo Fisher Scientific) equipped with a heated ESI source and operating in Full-ms/ddMS^2^ mode.

### 2.3 Lithium-pilocarpine hydrochloride-induced epileptic rat model

SD rats (180–220 g) were purchased from SLAC Laboratory Animal Co., Ltd. (Shanghai, China), and housed under specific pathogen-free (SPF) conditions at the laboratory animal center of the First Affiliated Hospital of Zhengzhou University, with 22 ± 2°C temperature, 50% humidity, 12 h light/dark cycle, and *ad libitum* access to food and water. According to clinical application, the dosage of XYC and CBZ is 6.6 g and 1.2 g per person per day, respectively. Based on the body surface area normalization from rodent-to-human equivalent dose conversion, the equivalent dose for rat is 0.70 g/kg and 0.125 g/kg per day. Thirty rats were randomly assigned to five groups (Control, Model, CBZ, XYC 0.35 g/kg and 0.70 g/kg group) using a computer-generated block randomization scheme (block size = 6) to ensure balanced distribution of body weights and baseline metabolic parameters. Randomization was performed by an independent researcher not involved in data collection, and group allocation was concealed until interventions began. After 7 days of adaptive feeding, the rats were administered with CBZ or XYC by gavage for 14 days. The dosage of CBZ was 0.125 g/kg, the high dosage of XYC was 0.70 g/kg, and the low dosage was 0.35 g/kg. On the 15th day, epilepsy was induced as previously described (34, 35). Following an initial intraperitoneal (*i.p.*) administration of lithium chloride (3 mmol/kg), rats received pilocarpine (35.3 mg/kg, *i.p.*) 20 h post-injection. Seizures manifested 15–35 min post-induction and were quantified over 30 min using the established modified Racine scale (36). The seizures were terminated with 10% chloral hydrate saline solution 1 h after the seizures. The epileptic rats were housed with food and water available *ad libitum*. After 7 days of continuous intragastric administration, the rats were sacrificed, blood was collected for metabonomic detection. The cecal contents were collected for 16S rDNA sequencing. The hippocampal tissues were collected for the determination of MDA, SOD and pathological observation. Throughout the experiment, investigator blinding and outcome assessor blinding was implemented. Ethical approval (2023-KY-1341) was granted by the Research Ethics Committee at Zhengzhou University First Hospital, with informed consent obtained following NIH guidelines for animal welfare.

### 2.4 Biochemical analysis

The catalase activity analysis was added here: “Catalase (CAT) activity was quantified by measuring the absorbance at 405 nm of a yellow-colored complex formed between residual H₂O₂ and ammonium molybdate, which reflects the remaining H₂O₂ after catalase-mediated decomposition.”

### 2.5 ELISA

The serum concentrations of proinflammatory cytokines (IL-6, TNF-*α*, and IL-1β) were determined via ELISA according to manufacturer protocols. Absorbance measurements at 450 nm were performed on a Varioskan LUX multimode microplate reader.

### 2.6 Hematoxylin and eosin and Nissl staining

Following intervention, hippocampal specimens were immediately immersion-fixed in 4% paraformaldehyde. Gradient ethanol dehydration preceded paraffin embedding. Serial coronal sections (5 μm thickness) were obtained for subsequent histological processing, including hematoxylin & eosin (H&E) and cresyl violet-based Nissl staining (37). Histopathological scoring was performed by two independent pathologists blinded to treatment conditions.

### 2.7 16S rDNA sequencing

The isolation of total DNA from intestinal microbiota, followed PCR reaction and sequencing were performed as described before (38). RDP classifier^1^ was used to annotate the representative sequences after clustering, and a threshold of 70% was set against the Silva database (SSU128/16 s bacteria). Using the R language tool to draw the community histogram, we determined the structural composition of different groups of the bacterial community at the phylum and generic levels.

### 2.8 Ultra high performance liquid chromatography quadrupole time-of-flight mass spectrometry for untargeted metabolomics in serum

For metabolomics analysis, serum were collected after treatment. The chromatographic conditions in UHPLC system and the QExactive high-resolution system for mass spectrometry systems were conducted as previously described (39, 40).

Briefly, Serum were vortexed with methanol/acetonitrile (1:1, v/v), centrifuged (4°C 14,000 × g for 20 min), and the resultant supernatant lyophilized before reconstitution in acetonitrile/water (1:1, v/v).

UHPLC system (Vanquish, Thermo) with HILIC column were used. Mobile phase: A = 25 mM ammonium acetate/hydroxide, B = acetonitrile. Gradient: 98% B (1.5 min) to 2% B (10.5 min), held 2 min, rapidly restored to 98% B (0.1 min) with 3 min re-equilibration in both ESI modes.

Processed raw MS data (ProteoWizard) using XCMS with centWave (peakwidth = 10–60, m/z = 10 ppm) and grouping parameters (mzwid = 0.025, bw = 5). CAMERA annotated isotopes/adducts. Metabolites were identified via m/z (<10 ppm) and MS/MS matching against an authentic standards database. Normalized data underwent multivariate analysis (Pareto-scaled PCA/OPLS-DA) via ropls. Model robustness was verified by 7-fold cross-validation/permutation tests.

### 2.9 Network pharmacology analyze

Bioactive constituents of XYC’s constituent herbs were systematically curated from the Traditional Chinese Medicine Systems Pharmacology (TCMSP, https://tcmspw.com/tcmsp.php) or BATMN-TCM^2^ using the keywords “*Hedysarum Multijugum* Maxim.,” “Codonopsis Radix,” “Radix Salviae,” “Radix Bupleuri,” “Ziziphi Spinosae Semen,” “Polygala tenuifolia Willd.,” “Rhizom Gastrodiae,” “Uncariae Ramulus Cumuncis,” “Acoritataninowii Rhizoma,” “Arisaema *Cum* Bile,” “Angelicae Sinensis Radix,” “Bombyx Batryticatus,” “Massa Medicata Fermentata,” “Curcumae Radix,” “licorice” and “Typhonii Rhizoma.” Then screen the retrieved compounds by ADME parameters (oral bioavailability (OB) ≥ 30%, drug-likeness (DL) ≥ 0.18) (41). Epilepsy-related disease targets were obtained from OMIM,^3^ DisGeNET,^4^ and GeneCards databases.^5^ Then XYC-epilepsy-related target screening and PPI network construction were obtained as previously described (42).

### 2.10 RT-qPCR

Total RNA from hippocampal tissues were isolated using TRIzol and reversed into cDNA. RT-qPCR was conducted using SYBR qPCR Master Mix (#A57155, Applied Biosystems, MA, United States). Primers sequences were listed in Table 1.

**Table 1:** Primer sequences used in RT-qPCR.

Target	Forward (5′-3′)	Reverse (5′-3′)
*Il-6*	CACTTCACAAGTCGGAGGCT	TCTGACAGTGCATCATCGCT
*Ins*	CAGGACAGGCTGCATCAGAA	TTCCCCGCACACTAGGTAGA
*Akt1*	ATGAACGACGTAGCCATTGTG	TTGTAGCCAATAAAGGTGCCAT
*Gapdh*	ACGGGAAACCCATCACCATC	ACGACATACTCAGCACCAGC

### 2.11 Western blotting assay

Hippocampal protein was extracted using ice-cold RIPA lysis buffer (#R0010, Solarbio) supplemented with 1% protease/phosphatase inhibitor cocktail (#P1260, Solarbio, Beijing, China), and then separated with SDS-PAGE, and PVDF membranes were used for the incubation of primary antibodies followed by secondary antibodies, and ECL substrate for visualizing the results.

### 2.12 Statistical analysis

Statistical analyses were conducted with SPSS Statistics (v19.0, IBM Corp.) using the mean ± standard error of mean (SEM). The significance between three or more groups was determined by one-way ANOVA, with *p* < 0.05 considered statistically significant.

## 3 Results

### 3.1 Chemical components of XYC

**Figure 1:**
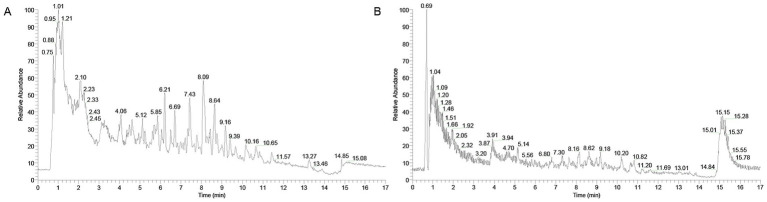
Total ion chromatograms of XYC in negative **(A)** and positive **(B)** ion modes.

The chemical constituents of XYC were identified by UPLC-MS analysis. Our study revealed that XYC contains abundant constituents, including organic oxygen compounds, phenylpropanoids and polyketides, benzenoids, organoheterocyclic compounds and lipids and lipid-like molecules. A total of 904 compounds were identified, the positive and negative ion flow chromatography were shown in Figure 1 and the top ten anionic and cationic constituents, ranked by peak area, were presented in Table 2.

**Table 2:** Characterization of components in XYC by UHPLC Q/Exactive HFX MS.

No.	Metabolite	Formula	Rt/min	Mass Error (ppm)	m/z	Mode	Adducts	Pubchem_ID	Class
1	Parishin E	C_19_H_24_O_13_	4.06	−1.25	459.11	neg	M-H	91,973,797	Organooxygen compounds
2	Salvianolic acid A	C_26_H_22_O_10_	6.71	−1.51	493.11	neg	M-H, 2 M-H	5,281,793	Stilbenes
3	4-Hydroxyphenylpyruvic acid	C_9_H_8_O_4_	2.08	1.18	197.05	neg	M-H, M-H + H2O	979	Benzene and substituted derivatives
4	Danshensu	C_9_H_10_O_5_	2.08	−2.26	395.10	neg	2 M-H	11,600,642	Phenylpropanoic acids
5	Citric acid	C_6_H_8_O_7_	0.95	−1.85	191.02	neg	M-H		
6	Parishin C	C_32_H_40_O_19_	4.94	−0.76	727.21	neg	M-H	10,676,408	Organooxygen compounds
7	3-Furfuryl 2-pyrrolecarboxylate	C_10_H_9_NO_3_	2.23	−2.15	236.06	neg	M + HCOO	189,695	Pyrroles
8	Cryptochlorogenic acid	C_16_H_18_O_9_	3.62	−1.77	353.09	neg	M-H	9,798,666	Organooxygen compounds
9	Licoricesaponin G2	C_42_H_62_O_17_	8.04	−0.89	837.39	neg	M-H	14,891,565	Prenol lipids
10	Neoagarobiose	C_12_H_20_O_10_	1.08	−2.19	323.10	neg	M-H, 2 M-H	54,758,702	Organooxygen compounds
11	Dihydroobovatin	C_20_H_20_O_4_	5.16	−0.81	342.17	pos	M + NH4	73,554,083	Flavonoids
12	glycocholic acid	C_26_H_43_NO_6_	8.16	−0.79	430.29	pos	M + H-2H2O, M + H, M + Na, 2 M + H, M + K, M + H-H2O, M + NH4	10,140	Steroids and steroid derivatives
13	Hirsuteine	C_22_H_26_N_2_O_3_	7.65	−0.83	367.20	pos	M + H		
14	Maltol	C_6_H_6_O_3_	1.98	−4.81	145.05	pos	M + H2O + H	8,369	Pyrans
15	Corynoxeine	C_22_H_26_N_2_O_4_	6.55	−1.11	383.20	pos	M + H	10,475,115	Indolizidines
16	Hypoxanthine	C_5_H_4_N_4_O	1.20	−3.82	136.06	pos	M + NH4-H2O	135,398,638	Imidazopyrimidines
17	Octadecyl caffeate	C_27_H_44_O_4_	11.22	−2.00	496.34	pos	M + CH3CN + Na	5,320,237	Cinnamic acids and derivatives
18	Allo-Yohimbine	C_21_H_26_N_2_O_3_	7.26	−1.01	355.20	pos	M + H		
19	8-Acetyl-7-Hydroxycoumarin	C_11_H_8_O_4_	7.43	7.51	309.09	pos	M + 2CH3CN + Na	5,411,574	Coumarins and derivatives
20	Citrulline	C_6_H_13_N_3_O_3_	1.00	−0.30	158.09	pos	M + H-H2O	9,750	Carboxylic acids and derivatives

### 3.2 XYC treatment alleviated seizure in lithium-pilocarpine-induced acute epilepsy rat model

**Figure 2:**
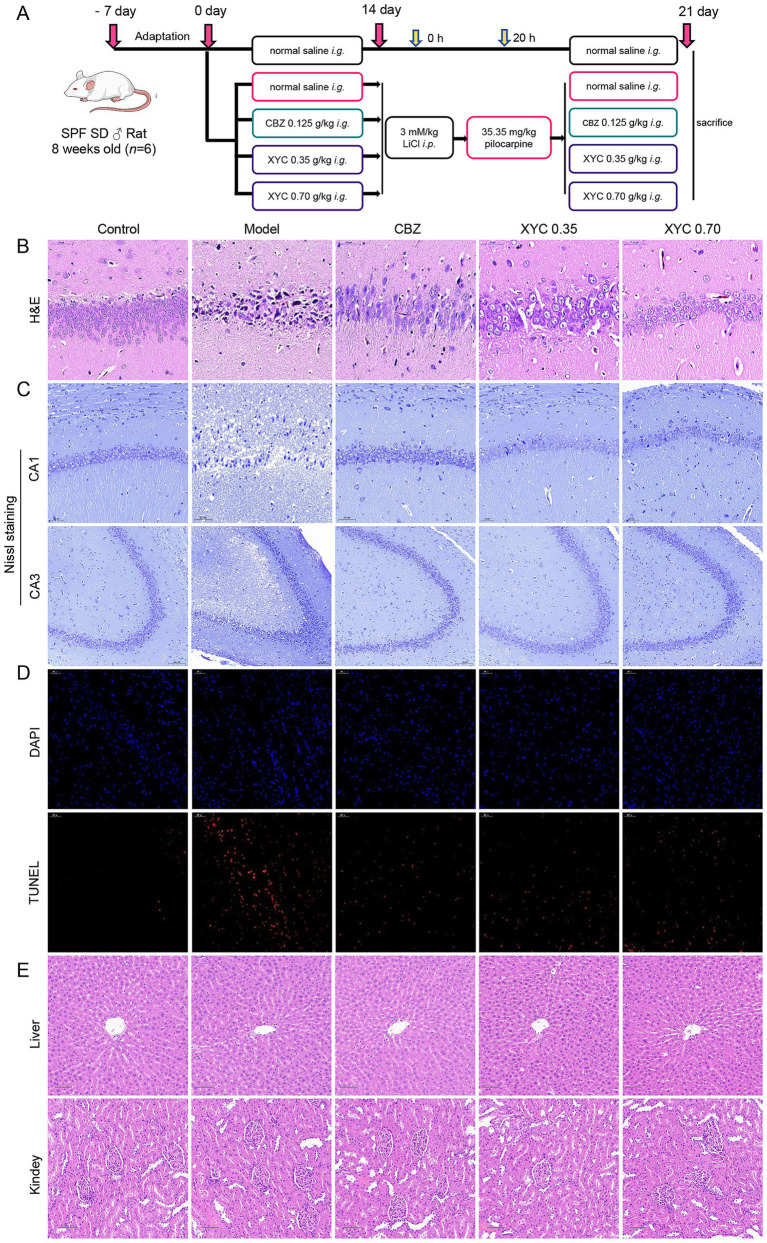
XYC treatment alleviated seizure in lithium-pilocarpine-induced acute epilepsy rats. **(A)** Schematic of the rat epilepsy model construction and XYC administration. **(B–D)** Hippocampus CA1 region tissues were collected and the histological changes were assessed using H&E and Nissl staining. **(B)** Representative images of H&E staining (Scale bar = 50 μm). **(C)** Representative images of Nissl staining (Scale bar = 50 μm in CA1 region, Scale bar = 100 μm in CA3 region). **(D)** Representative images and quantitative analysis results of TUNEL staining (Scale bar = 50 μm). **(E)** Pathological changes in the liver and kidney tissues were examined with H&E staining (scale bar = 100 μm).

To delineate XYC’s anti-epileptic pharmacodynamics, we established a lithium-pilocarpine rat model of acute epileptogenesis. A schematic overview of the treatment regimen and experimental workflow is presented in Figure 2A. Treatment with CBZ and XYC significantly prolonged the seizure latency and reduced the seizure frequency induced by pilocarpine in rat models of epilepsy (Table 3). H&E staining results found that, the hippocampal CA1 region tissues in the control group exhibited normal cellular morphology with neatly arranged cells, abundant cytoplasm, and large, round nuclei. In contrast, the model group showed a noticeable reduction in cell count, accompanied by a loose and disorganized arrangement. The cytoplasm appeared deeply stained, with evident nuclear dissolution and fragmentation, along with vacuolated manifestations. Both the CBZ and XYC groups demonstrated improved hippocampal cellular characteristics compared to the model group. Specifically, these groups exhibited increased cell density, gradual restoration of normal cellular morphology, and better-organized cellular alignment. Furthermore, the pathological manifestations of nuclear fragmentation and pyknosis were significantly alleviated (Figure 2B).

**Table 3:** Effect of XYC on lithium-pilocarpine-induced acute epilepsy determined as seizure latency and numbers of seizure.

Group	Dose (g/kg)	Latency to the first seizures (min)	Numbers of seizure
Control	–	–	–
Model	–	23.12 ± 8.28	2.37 ± 0.23
CBZ	0.125	43.62 ± 10.85^#^	1.15 ± 0.12^#^
XYC	0.35	34.30 ± 7.31^*^	1.57 ± 0.24^*^
XYC	0.70	44.84 ± 8.13^*^	1.09 ± 0.17^*^

As shown in Figure 2C, Nissl staining revealed morphological changes of hippocampal CA1 neurons. Neurons in control group showing neatly arranged with intact morphology and clearly discernible Nissl bodies. While, model group exhibited a significant neuronal loss accompanied by disorganized cellular alignment and enlarged intercellular spaces. However, the CBZ and XYC treatment groups exhibited partial preservation of neuronal architecture in the hippocampal CA1 region, though with distinct pathological features. While neurons in these groups maintained relatively intact cellular morphology with preserved structural integrity, mild disorganization in cellular alignment was observed. Notably, Nissl body staining intensity demonstrated moderate attenuation, coupled with discernible enlargement of intercellular spaces. Despite these morphological alterations, the majority of neurons retained detectable viability and functional capacity. Quantitative analysis of TUNEL-positive cells combined with nuclear counterstaining revealed that both CBZ and XYC treatments significantly reduced apoptosis in CA1 neurons compared to the Model group (Figure 2D). Furthermore, the H&E staining of the liver and kidneys of each group were observed, and found that CBZ and XYC treatment did not changed the tissue morphology and showing no toxicity on liver and kidney (Figure 2E). These consistent findings across multiple staining techniques demonstrate that XYC pretreatment effectively mitigates seizure-induced neuronal injury in the hippocampal CA1 region.

### 3.3 Effect of XYC on lipid peroxidation and inflammation in lithium-pilocarpine-induced acute epilepsy rats

**Figure 3:**
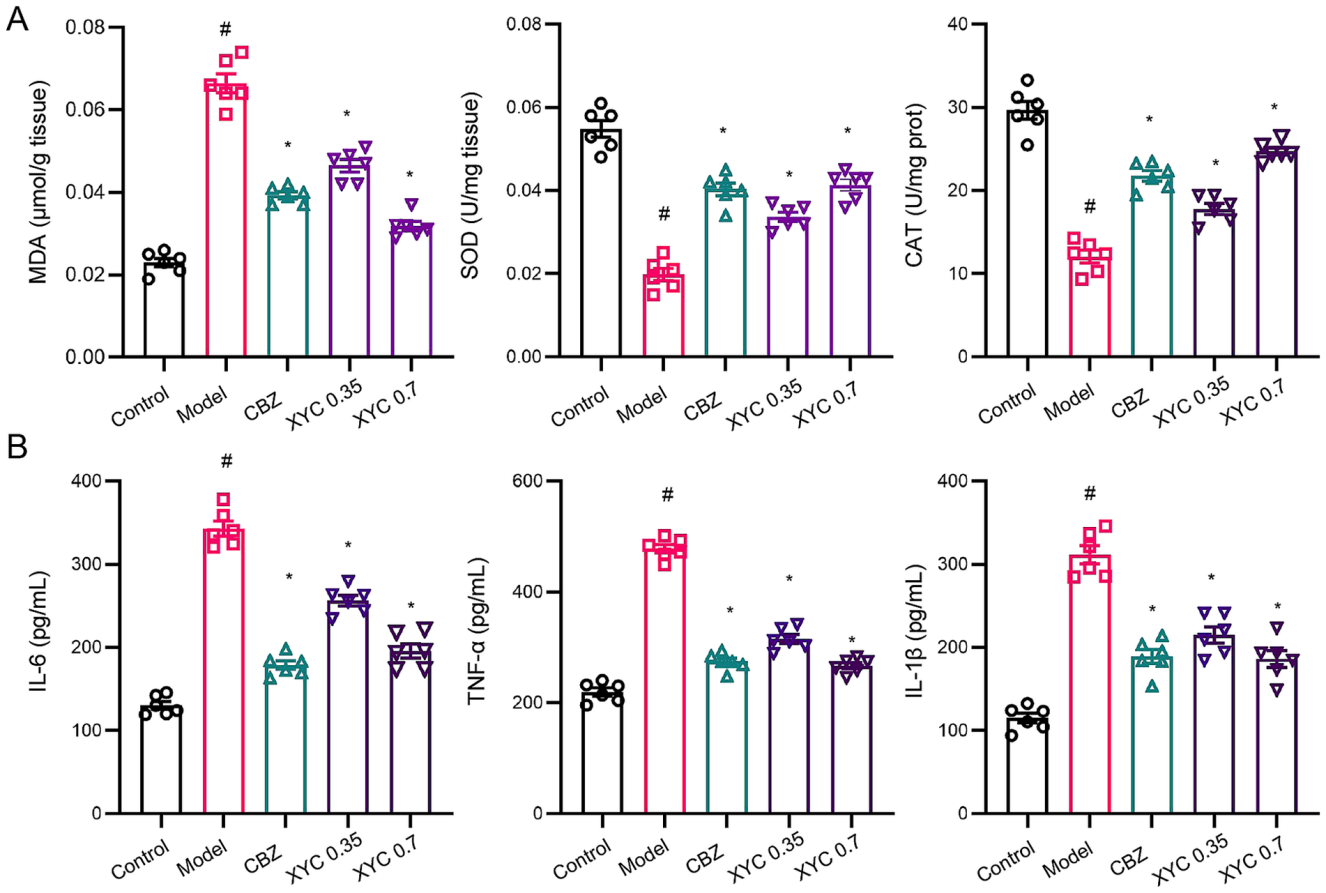
XYC treatment reduced lipid peroxidation and inflammation response in lithium-pilocarpine-induced acute epilepsy rats. **(A)** Effects of CBZ (0.125 g/kg) and XYC (0.35 and 0.70 g/kg) on lipid peroxidation and oxidative/nitrosative stress, specifically MDA, SOD and CAT in the hippocampus tissues. **(B)** Effect of CBZ and XYC on serum IL-6, TNF-*α* and IL-1β levels. Data are shown as mean±SEM (*n* = 6). ^#^ *p* < 0.05 vs. Control group; ^*^ *p* < 0.05 vs. Model group.

Neuroinflammation and oxidative stress represent pivotal mechanisms underlying epileptogenesis, with their pathophysiological contributions being linked to elevated disease susceptibility (43). Consequently, interventions that targeting these dual pathological processes may provide effective therapeutic strategies for mitigating epileptogenesis. As shown in Figure 3, biochemical analyses revealed that epilepsy induction significantly elevated MDA levels while reducing SOD activity and CAT content in hippocampal tissues, indicating pronounced oxidative stress. Both CBZ and XYC treatments effectively attenuated these pathological changes, demonstrating significant reductions in MDA content and restoration of SOD activity and CAT contents compared to untreated epileptic controls (Figure 3A). Furthermore, ELISA assay found that IL-6, TNF-*α* and IL-1β pro-inflammation cytokine levels were significantly increased in epileptic rats, while both CBZ and XYC treatments effectively reversed this upward trend (Figure 3B). These findings demonstrate that XYC treatment not only induced anti-oxidant but also anti-inflammatory properties in epilepsy rats.

### 3.4 Effect of XYC on gut microbiota profiles in lithium-pilocarpine-induced acute epilepsy rats

Emerging research has revealed that sophisticated interactions between gut microbiota and cerebral functions are mediated through an integrated network of cellular signaling mechanisms and neural communication circuits, collectively termed the “bidirectional microbiota-gut-brain axis” (44). Elucidating the microbiota’s dual role in both epileptogenesis processes and anticonvulsant therapeutic interventions could fundamentally advance our understanding of the neurobiological foundations underlying seizure disorders.

**Figure 4:**
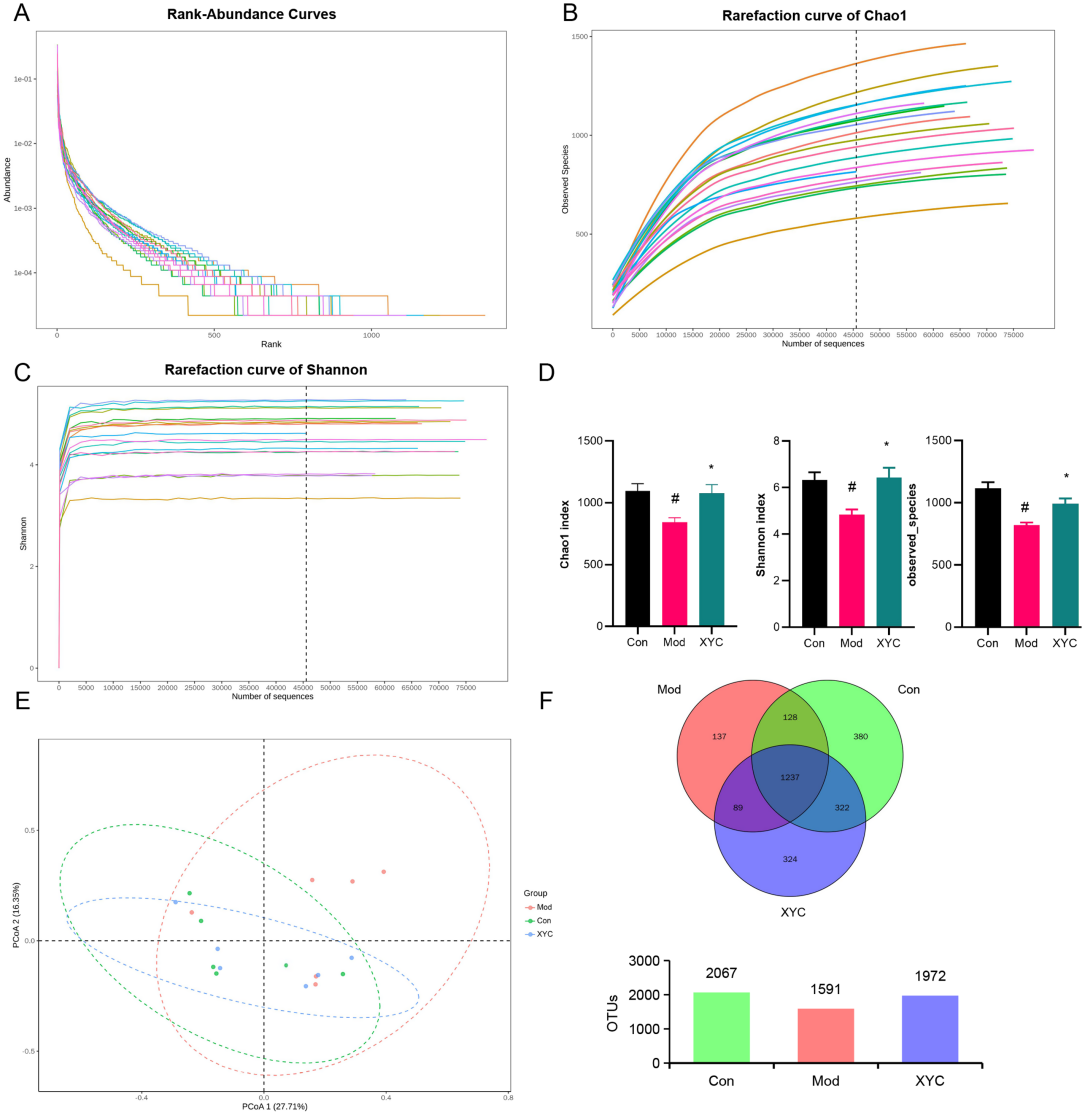
XYC improved the composition of the gut microbiota in lithium-pilocarpine-induced acute epilepsy rats. **(A)** Rank-abundance curves analysis at the OTU classification level. **(B)** Rarefaction curve of Chao1. **(C)** Dilution curve analysis: the Shannon index of the sample sequence at the OTU level. **(D)** Alpha diversity includes the Chao1 index, Shannon indices, and Observed OTUS. **(E)** PCoA obtained from LEfSe analysis. **(F)** Venn diagram analysis: counting the number of shared and unique OTUs in each group. Data are shown as mean±SEM (*n* = 6). ^#^ *p* < 0.05 vs. Control group; ^*^ *p* < 0.05 vs. Model group.

To elucidate the impact of XYC on intestinal homeostasis, 16S rDNA were amplified followed by bioinformatic analysis. Alpha diversity analysis (Chao1 index, Shannon index, and Observed OTUs) demonstrated significant microbial community changes following epilepsy induction and XYC treatment. While rank abundance curves and diversity indices showed comparable bacterial composition patterns among Control, Model, and XYC 0.7 groups (Figures 4A–C), quantitative analysis revealed distinct differences. The Model group exhibited significantly reduced species richness (Chao1 index, *p* < 0.05) compared to controls, consistent with established microbiota depletion in epilepsy (45). XYC treatment effectively restored microbial diversity, significantly increasing both the Chao1 index and Shannon diversity (*p* < 0.05 versus Model group, Figure 4D). Beta diversity analysis using PLS-DA demonstrated clear separation of gut microbiota communities among the three groups (Figure 4E). Venn diagram analysis at the OTU level identified 2067, 1,591, and 1972 operational taxonomic units in Control, Model, and XYC groups respectively, revealing both shared and group-specific microbial signatures (Figure 4F).

**Figure 5:**
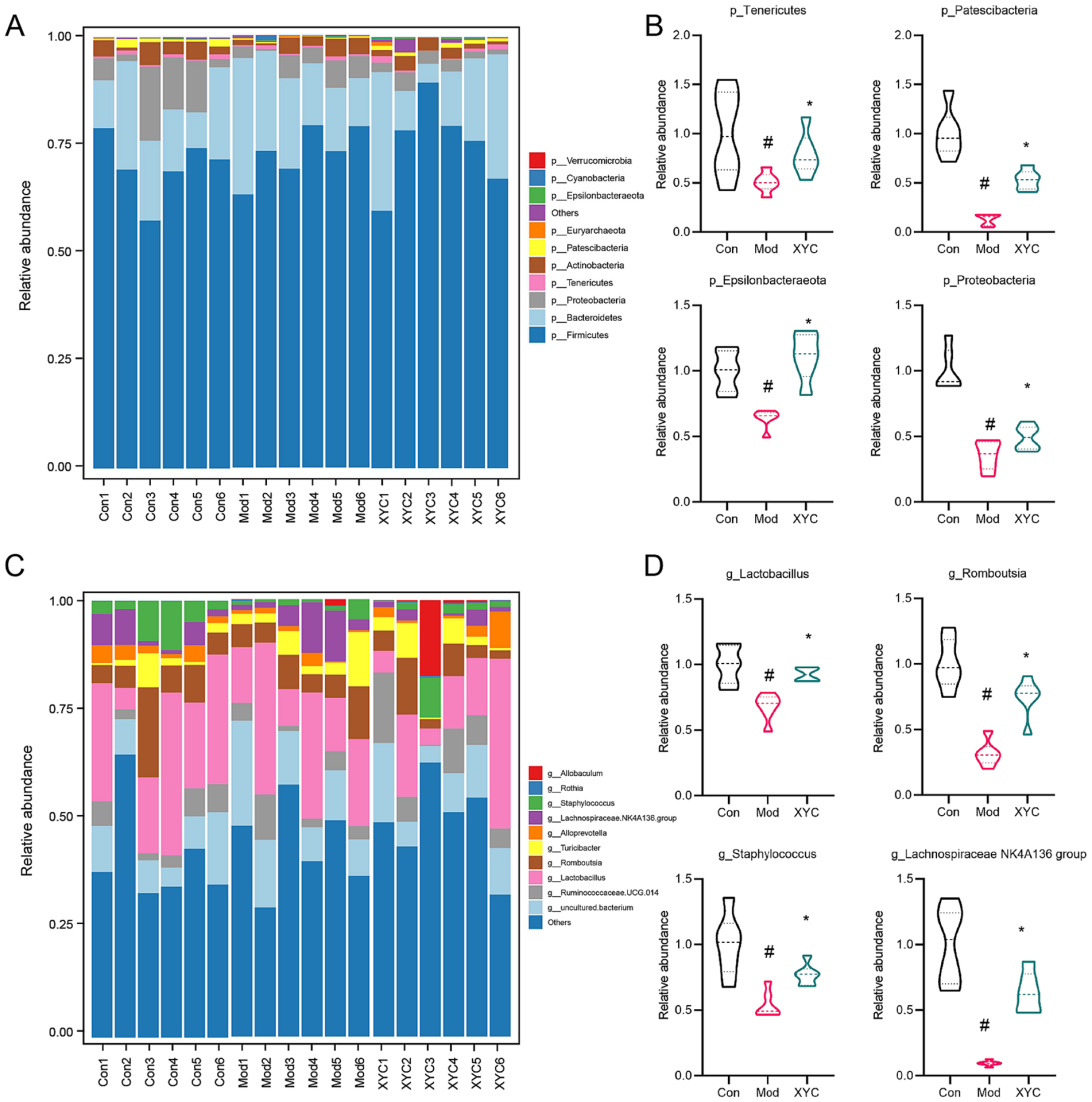
XYC treatment altered the gut microbiota disturbance in lithium-pilocarpine-induced acute epilepsy rats. **(A,B)** Dominant phylum-level microbiota distribution (top 10 taxa) was compared between groups, while genus-specific abundance variations among each group were quantified. **(C,D)** Dominant genus distributions (top 10 taxa) were compared between groups, while LEfSe analysis identified differential abundance patterns among each group. Data are shown as the mean±SEM (*n* = 6). ^#^ *p* < 0.05 compared to the Control group; ^*^ *p* < 0.05 compared to the Model group.

Microbiome profiling demonstrated marked taxonomic restructuring of gut microbial architecture across phylum and genus hierarchies post-epileptogenesis and XYC intervention. At the phylum level, epileptic rats exhibited marked reductions in *Tenericutes*, *Patescibacteria*, *Epsilonbacteraeota*, and *Proteobacteria* abundance compared to controls (*p* < 0.05). XYC treatment substantially restored these depleted phyla to near-normal levels (Figures 5A,B). Genus-level analysis demonstrated that XYC effectively counteracted epilepsy-induced dysbiosis, significantly preserving populations of beneficial taxa including *Lactobacillus*, *Ramboutsia*, *Staphylococcus*, and *Lachnospiraceae NK4A136 group* (Figures 5C,D).

**Figure 6:**
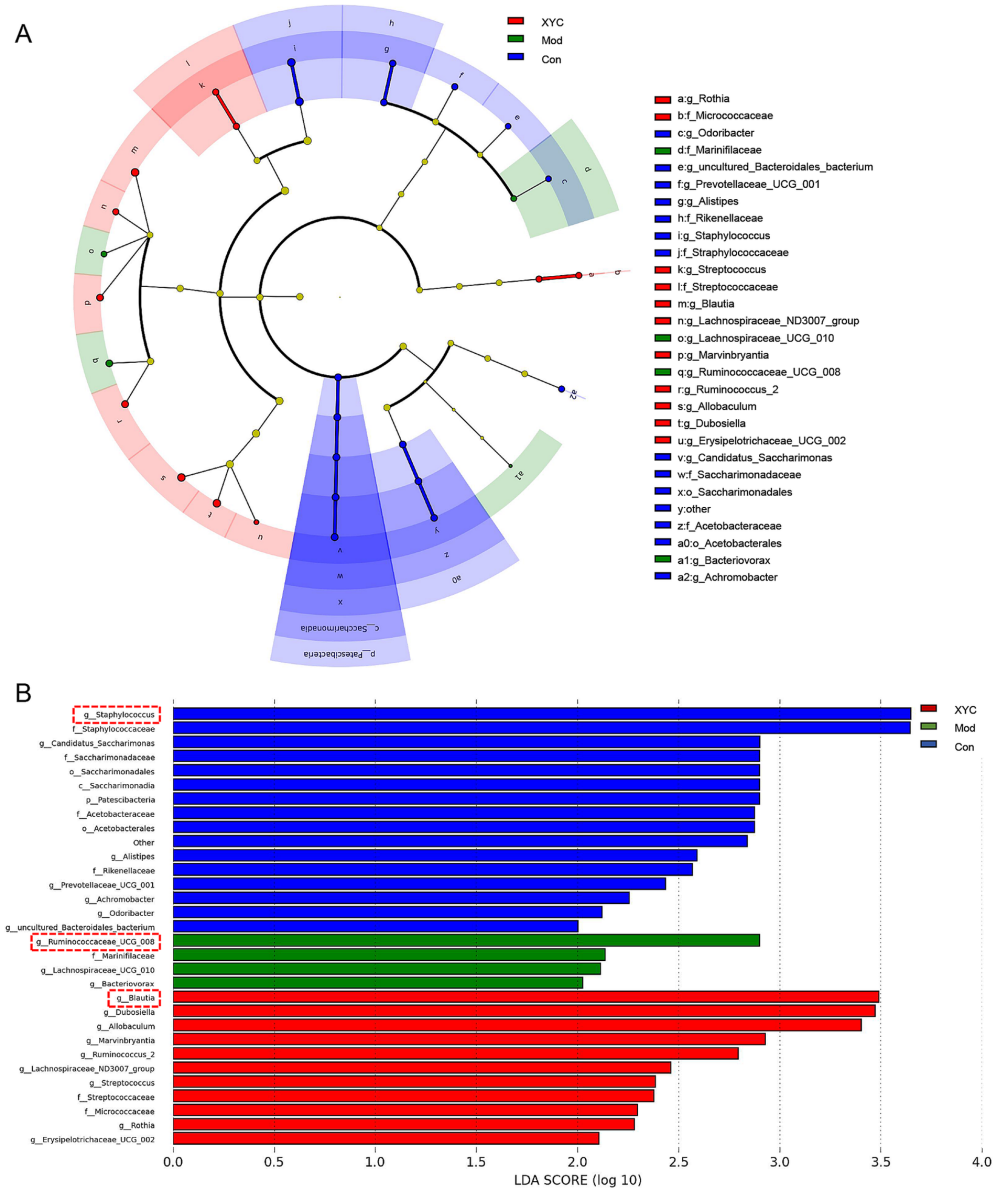
Taxonomic biomarkers identification via LEfSe. **(A)** Cladogram illustrating phylogenetic distribution of discriminative taxa across three experimental groups, with radiating circles denoting taxonomic hierarchy. Node diameters reflect relative abundance. **(B)** Linear discriminant analysis (LDA) scores highlight group-specific biomarkers.

Microbial community differences among the three experimental groups were visualized through LEfSe analysis, generating a cladogram that highlights taxonomically distinct features at multiple phylogenetic levels (Figure 6A). Using stringent criteria (LDA score > 2, *p* < 0.05), we identified group-specific microbial signatures: *Staphylococcus, Ruminococcaceae_UCG_008* and *Blautia* abundance characterized the Control, Model and XYC group, respectively (Figure 6B).

### 3.5 XYC altered serum metabolites in lithium-pilocarpine-induced acute epilepsy rats

**Figure 7:**
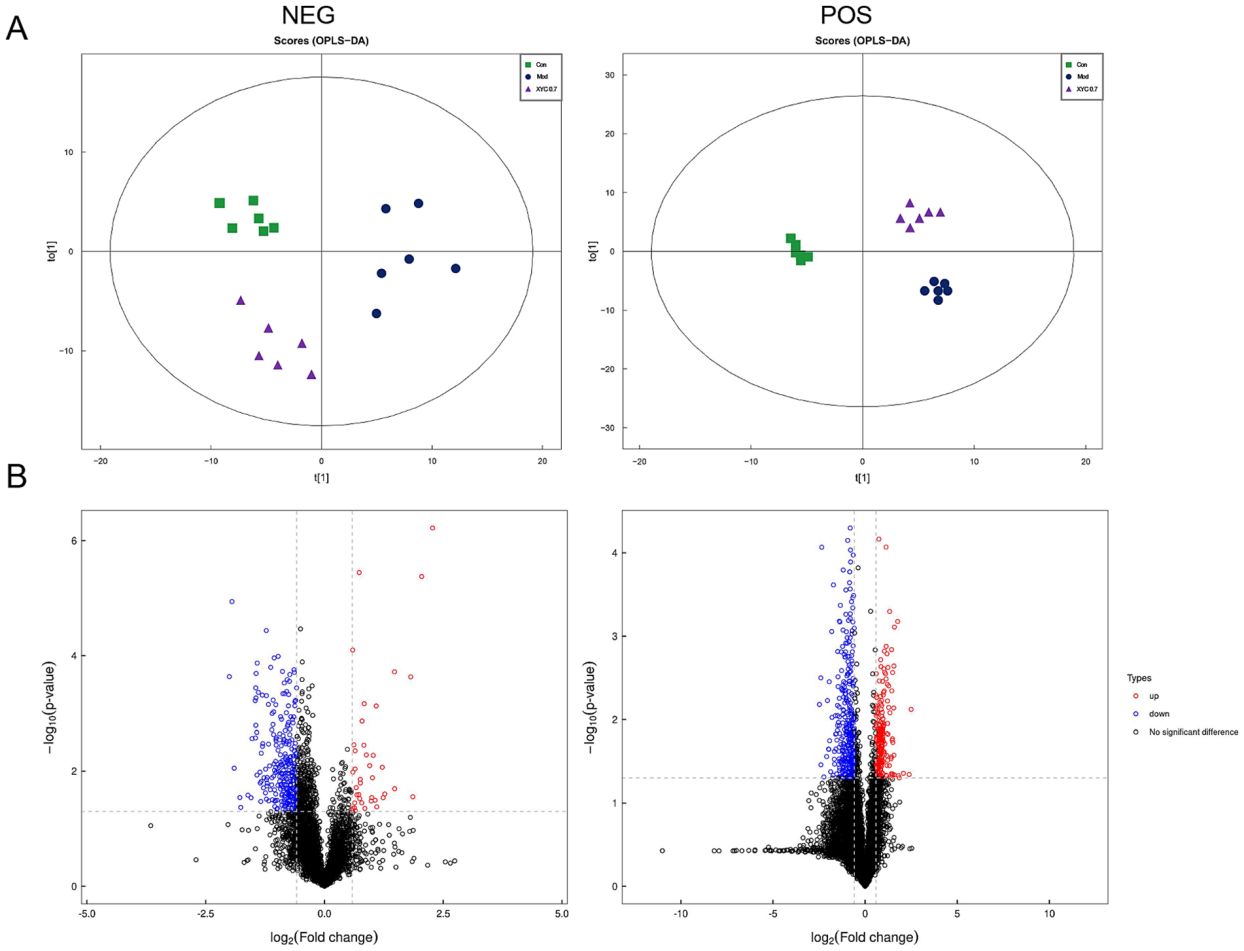
XYC treatment altered the serum metabolites in lithium-pilocarpine-induced acute epilepsy rats. **(A)** OPLS-DA of the negative and positive ion mode. **(B)** Volcano plot of the differential metabolites.

Serum metabolite profiling was conducted using UHPLC-QTOF-MS/MS, revealing distinct metabolic patterns among experimental groups. OPLS-DA demonstrated clear separation among groups (R^2^X = 0.35, R^2^Y = 0.952, Q^2^ = 0.512 for negative ion mode, R^2^X = 0.566, R^2^Y = 0.993, Q^2^ = 0.532 for positive ion mode, Figure 7A). Using stringent criteria (VIP > 1, *p* < 0.05), we identified 970 significantly altered metabolites. Metabolomic profiling showed 157 differentially expressed metabolites between groups (FC ≥ 2 or ≤ 0.5, *p* < 0.05), with Model specimens exhibiting 73 upregulated versus 84 downregulated species relative to Controls. Notably, XYC treatment modulated 149 metabolites compared to the Model group, including 72 elevated and 77 reduced species (Figure 7B). Hierarchical clustering analysis and fold-change distributions further illustrated these metabolic shifts (Supplementary Figures S1, S2).

### 3.6 Regulation of XYC on the glycerophospholipid metabolism pathway of epilepsy rats

**Figure 8:**
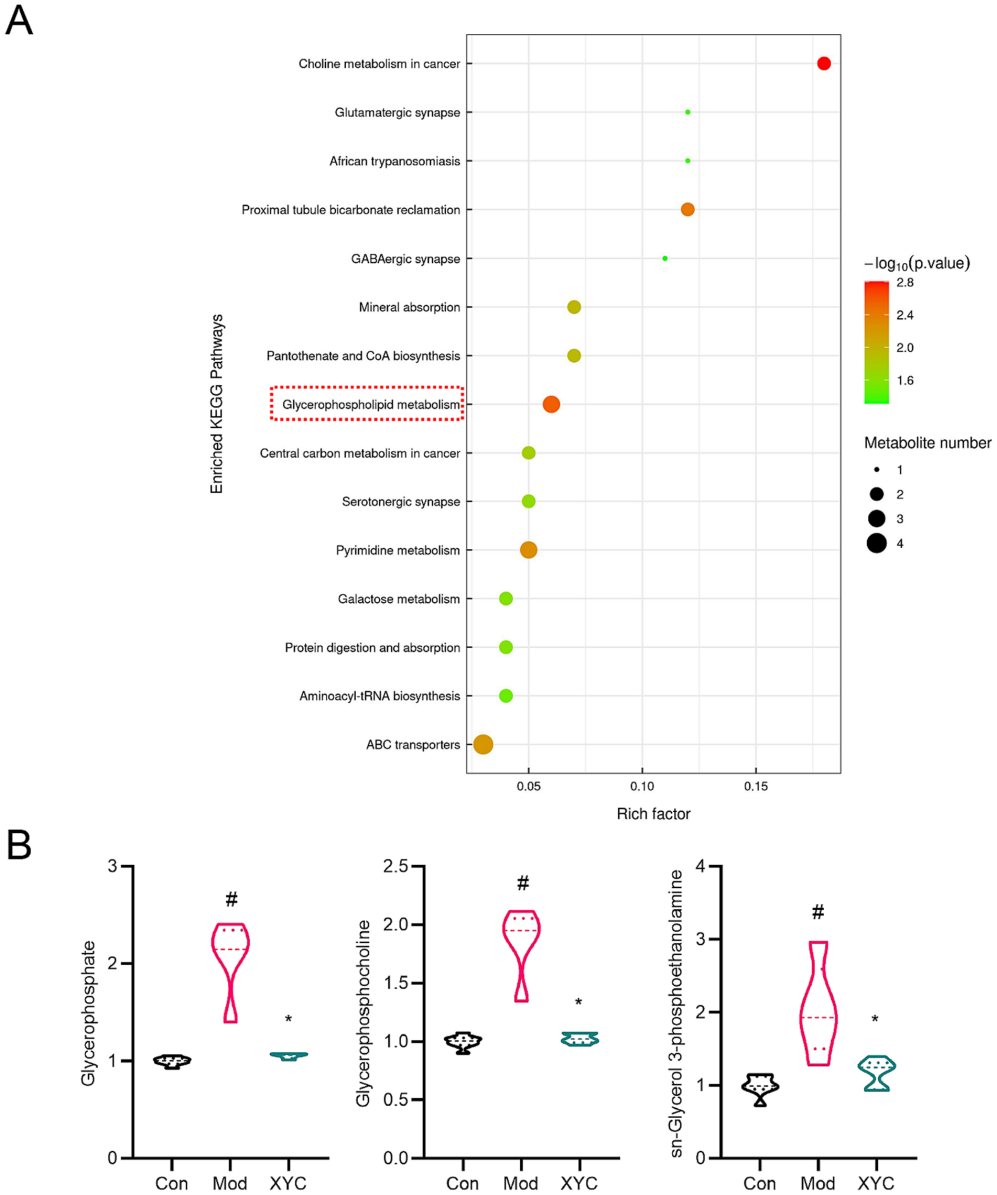
XYC altered the metabolites levels in glycerophospholipid metabolism pathway. **(A)** KEGG analysis of differential metabolites. **(B)** Diagram of relative abundance of different metabolites in serum. Data are shown as mean±SEM (*n* = 6). ^#^*p* < 0.05 vs. Control group; ^*^p < 0.05 vs. Model group.

KEGG pathway enrichment analysis demonstrated that XYC treatment significantly modulated multiple metabolic pathways in epileptic rats (Figure 8A). The most affected pathways included glycerophospholipid metabolism, pyrimidine metabolism, ABC transporters, proximal tubule bicarbonate reclamation, choline metabolism, and mineral absorption. The relative content of metabolites in the glycerophospholipids pathway were shown in Supplementary Table S1. Targeted analysis of glycerophospholipid metabolism revealed three significantly elevated metabolites in the Model group compared to controls: glycerophosphate (log_2_FC = 1.05, *p* = 0.004), glycerophosphocholine (log_2_FC = 0.89, *p* = 0.003), and sn-glycerol-3-phosphoethanolamine (log_2_FC = 1.01, *p* = 0.021). XYC treatment effectively normalized these aberrant metabolite levels (*p* < 0.05 vs. Model group; Figure 8B).

### 3.7 Correlation between gut microbes and metabolites

**Figure 9:**
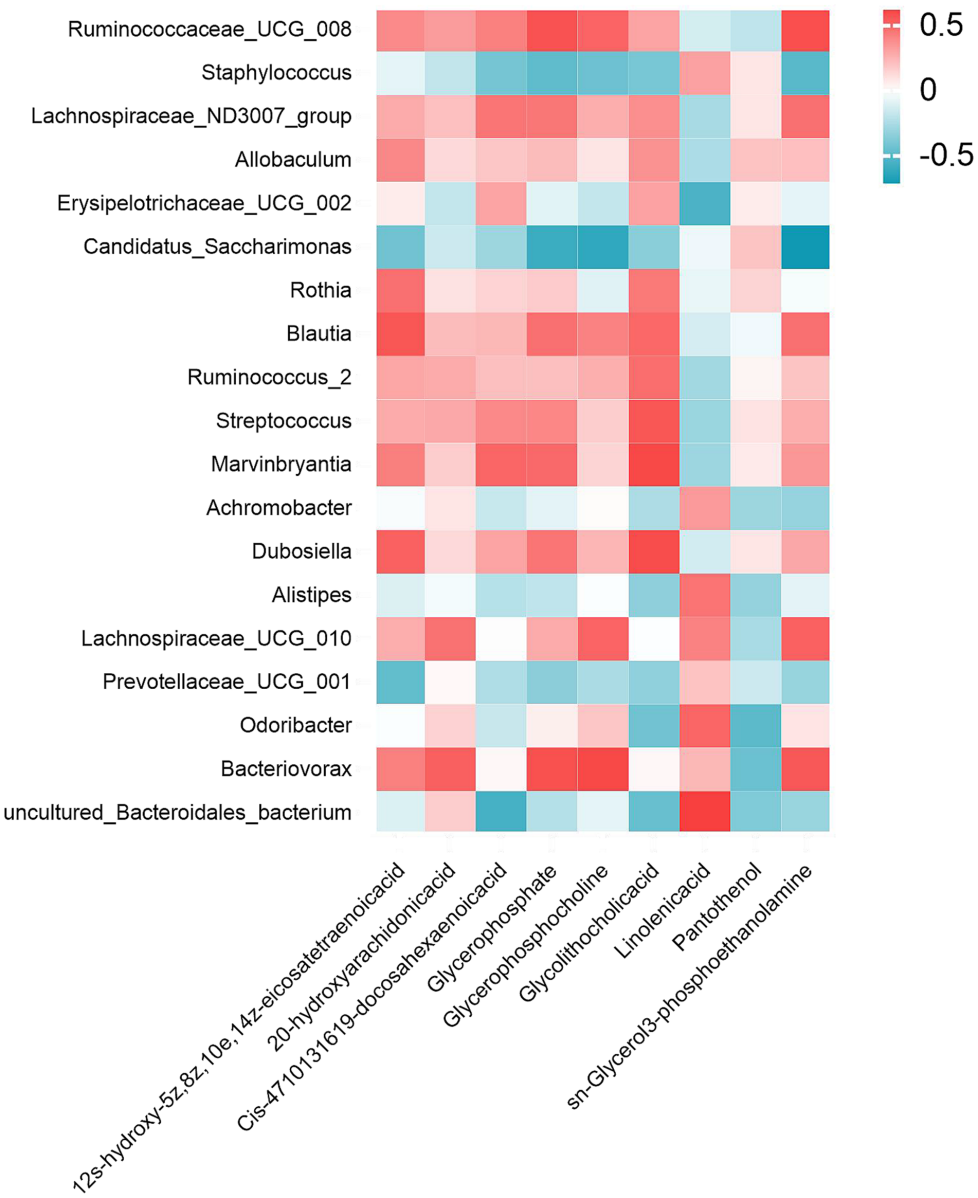
Gut microbiota-metabolite interaction network. Spearman correlation heatmap illustrating significant associations between the top 19 genus-level microbial taxa and 9 XYC modulated dysregulated metabolites. Red/blue hues denote positive/negative correlations.

To investigate potential microbiota-metabolite interactions, spearman correlation analysis was performed to examine associations between the 19 most abundant bacterial genera and 9 differentially expressed metabolites. Figure 9 delineates statistically robust associations between specific microbial taxa and glycerophospholipid metabolites: *Candidatus_Saccharimonas* exhibited negative correlations with glycerophosphate, glycerophosphocholine and sn-Glycerol 3-phosphoethanolamine; *Bacteriovorax* showed positive correlations with glycerophosphate and glycerophosphocholine; and *Ruminococcaceae_UCG_008* demonstrated a distinct pattern, correlating positively with sn-glycerol 3-phosphoethanolamine.

### 3.8 Network pharmacology of XYC against epilepsy

Using the TCMSP database, we systematically screened herbal components for the XYC formulation based on pharmacokinetic properties, while excluding compounds lacking biological targets. The analysis identified bioactive constituents from 16 medicinal herbs: Glycyrrhiza uralensis (88 compounds), *Salvia miltiorrhiza* (46), Astragalus membranaceus (17), Uncaria rhynchophylla (32), Bupleurum chinense (13), Gastrodia elata (14), Polygala tenuifolia (18), *Codonopsis pilosula* (16), *Ziziphus jujuba* var. spinosa (7), Acorus tatarinowii (4), along with fewer components from Angelica sinensis (2), Arisaema erubescens (3), Aconitum coreanum (3), *Curcuma aromatica* (3), Bombyx batryticatus (1), and Massa Medicata Fermentata (2). Target integration and deduplication yielded 773 unique protein targets, representing the potential therapeutic target space of the XYC formulation.

**Figure 10:**
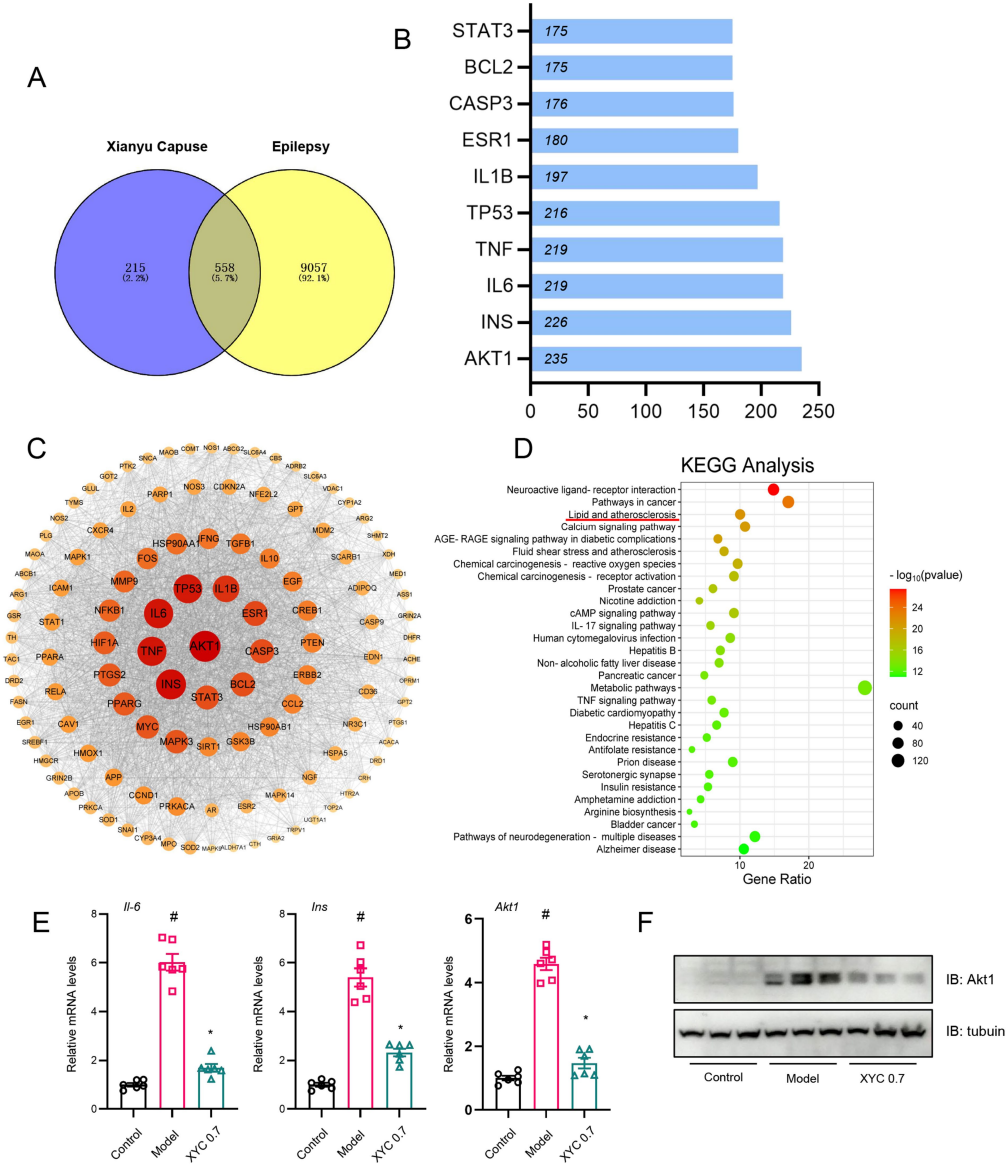
Network pharmacology analysis and validation of XYC. **(A)** Network pharmacology analysis revealed 558 overlapping nodes between XYC’s bioactive compounds and epileptogenesis-associated pathways, delineating therapeutic target convergence. **(B)** The PPI network of targets. **(C)** The ‘Compound-Target-Pathway-Disease’ network delineates polypharmacological interactions among XYC’s phytochemicals, epileptogenesis-associated targets, and dysregulated signaling axes. **(D)** KEGG pathway enrichment analysis of top 30 dysregulated metabolic routes, with lipid metabolism and inflammation showing highest significance. **(E)** *Il-6*, *Ins* and *Akt1* mRNA levels were quantified by RT-qPCR. **(F)** Hippocampal Akt1 protein levels were validated via immunoblotting. Data are shown as the mean±SEM (*n* = 6). ^#^*p* < 0.05 compared to the Control group; ^*^p < 0.05 compared to the Model group.

Through comprehensive integration of disease target databases (GeneCards, OMIM, and DisGeNET), we identified 9,615 epilepsy-associated targets, which were subsequently normalized using UniProt identifiers. Comparative analysis revealed 558 overlapping targets between these epilepsy-related genes and the XYC’s potential therapeutic targets (Figure 10A), representing the putative molecular targets through which the formulation may exert its anti-epileptic effects.

Ingredient-target network (283 active ingredients from XYC and 588 targets in epilepsy) was constructed with Cytoscape (version 3.10.3) (Supplementary Figure S3), and the PPI network was shown in Supplementary Figure S4. Topological analysis the top 10 targets as shown in Figure 10B, and the protein–protein interaction (PPI) network was constructed using Cytoscape, with the top 25 hub genes identified by node degree analysis (Figure 10C). KEGG pathway enrichment analysis of these hub genes revealed the top 30 significantly enriched pathways (Figure 10D), among which neuroactive ligand-receptor interactions, pathways in cancer, and lipid metabolism/atherosclerosis were most prominent. Notably, AKT1, INS, and IL-6 emerged as central nodes, which were functionally associated with lipid metabolic and inflammation processes, particularly glycerophospholipid metabolism, suggesting their potential as key therapeutic targets of XYC for epilepsy treatment.

To investigate the targets underlying the anti-epileptic effects of XYC, we performed RT-qPCR and Western blot analyses. RT-qPCR revealed elevated mRNA levels of *Il-6*, *Ins*, and *Akt1* in the hippocampal tissues of model groups compared to controls. Notably, XYC intervention significantly attenuated these increases (Figure 10E). Consistent with the mRNA findings, immunoblotting showed upregulated AKT1 protein expression in the epileptic models. Importantly, this elevated expression was normalized by XYC treatment (Figure 10F).

These findings suggest that XYC effectively alleviated seizure in lithium-pilocarpine-induced acute epilepsy by preserved neuro-inflammatory and glycerophospholipid metabolism.

## 4 Discussion

Epilepsy constitutes a persistent neurological condition characterized by unprovoked seizure episodes stemming from dysregulated neuronal hyperexcitability (47). Growing evidence implicates neuroinflammation triggered by brain injury in epileptogenesis. Preclinical and human studies consistently show post-seizure events like gliosis, elevated pro-inflammatory mediators, blood–brain barrier disruption, and activated inflammatory pathways. Disrupted lipid homeostasis also act as a potential contributor to epileptogenesis through multifaceted mechanisms involving neuroinflammation, membrane instability, and metabolic dysfunction (48). The complex metabolic network governing lipids-including TG, cholesterol fractions, and fatty acids—regulates critical neuronal processes ranging from membrane integrity to energy metabolism. Recent studies have identified that particular metabolic derangements, such as impaired fatty acid *β*-oxidation and aberrant cholesterol metabolism, demonstrate strong epidemiological associations with epilepsy susceptibility (49). This correlation appears particularly pronounced in pediatric populations with inborn disorder of fatty acid metabolism (50). So, understanding these processes identifies potential therapeutic targets and diagnostic/prognostic biomarkers for epilepsy.

TCM has served as a clinically validated therapeutic modality for epilepsy management through multimodal mechanisms, including neurotransmitter modulation, anti-inflammatory action, and neuroprotection (51). However, existing mechanistic research primarily focuses on neurotransmitter systems, immune dysfunction, and glial cell activity. Recently, accumulating evidence implicates gut dysbiosis and lipid metabolic disturbances in epileptogenesis (32, 52). Nevertheless, whether TCM interventions can ameliorate epilepsy by targeting these pathways remains systematically underexplored.

In this study, 16S rDNA sequencing revealed that XYC treatment significantly increased the relative abundance of four bacterial phyla (*Tenericutes*, *Patescibacteria*, *Epsilonbacteraeota*, and *Proteobacteria*) in epileptic rats. Intriguingly, *Patescibacteria*, a phylum found in human adipose tissue, may regulate fatty acid metabolism, proposing a novel gut-lipid-brain axis mechanism for XYC’s antiepileptic action (53, 54). At the genus level, XYC treatment effectively counteracted epilepsy-associated microbial alterations, significantly restoring the abundances of *Lachnospiraceae NK4A136 group*, *Lactobacillus*, *Staphylococcus,* and *Romboutsia*, which were diminished in epileptic rats. Notably, these genera play crucial roles in maintaining gut homeostasis, with *Lactobacillus* and *Romboutsia* being particularly recognized for their anti-inflammatory properties and short-chain fatty acid production. Previous clinical studies have identified a significant reduction in *Lactobacillus* abundance in epileptic patients (55). *Lactobacillus* species biosynthesize gamma-aminobutyric acid (GABA), the principal inhibitory neurotransmitter (56). Increased colonic GABA bioavailability demonstrates neurochemical coupling with central GABAergic tone, suggesting microbiota-mediated gut-brain signaling contributes to seizure pathophysiology (57). *Lactobacillus* species also play critical roles in lipid metabolism and obesity regulation by modulating gut microbiota composition to enhance food digestion and nutrient absorption (58, 59). Suppressing *Lactobacillus* proliferation attenuates intestinal lipid absorption and inhibits adipose deposition, highlighting its dual role in metabolic and neurological disease (59). Notably, *Romboutsia*, a SCFA producing genus, was significantly increased in abundance following XYC treatment, with its levels showing a positive correlation with seizure severity scores (46). These data demonstrate that XYC mediates seizure suppression exerts via bidirectional modulation of gut microbial ecology and systemic metabolomic networks, by restoring epilepsy-depleted beneficial genera (e.g., *Lactobacillus*, *Lachnospiraceae NK4A136*) while enhancing SCFA-producing taxa (*Romboutsia*) and reconciling lipid metabolism dysregulation.

The brain exhibits unique metabolic features characterized by high mitochondrial density, enrichment of polyunsaturated fatty acids (PUFAs), and elevated oxygen consumption, rendering it particularly susceptible to metabolic disturbances. These metabolic peculiarities underlie the growing recognition of metabolite-trait associations in neurological disorders. For instance, altered levels of glycerophosphocholines (GPCs)-key phospholipid metabolites involved in membrane integrity and neurotransmission-are linked to synaptic dysfunction in Alzheimer’s disease (60). Epileptic seizures are marked by transient surges in neuronal energy demand, necessitating dynamic metabolic adaptations. This cyclical interplay between energy expenditure and compensatory mechanisms underscores the pivotal role of metabolic dysfunction in epilepsy, a disorder primarily characterized by recurrent seizures. Notably, perturbations in glycerophospholipid metabolism have been implicated not only in epilepsy but also in neuropsychiatric disorders. Studies across murine, rodent, and non-human primate models of depression consistently report dysregulated glycerophospholipid profiles (61–63). For instance, D-ribose administration induces depressive-like behaviors in rodents by disrupting gut microbiota homeostasis, thereby altering glycerophospholipid metabolism (64). AnGong NiuHuang Pill ameliorates traumatic brain injury via modulation of glycerophospholipid metabolism (65). These findings position glycerophospholipid pathways as promising targets for metabolic intervention in neurological diseases, but its role in epileptogenesis and disease progression remains unelucidated.

In this study, we discovered that XYC administration significantly attenuated seizure severity and frequency in a rat model of epilepsy. Untargeted serum metabolomics profiling further identified XYC-mediated suppression of key intermediates in the glycerophospholipid metabolic pathway, with pronounced reductions in glycerophosphate, GPC, and sn-glycerol 3-phosphoethanolamine levels. These results indicate that XYC exerts its anti-seizure effects through normalizing hyperactivated glycerophospholipid metabolism, and reestablishing neurochemical homeostasis-potentially via restoring membrane phospholipid balance.

Integrated network pharmacology analysis identified 558 overlapping targets between XYC’s putative therapeutic targets and epilepsy-associated genes. AKT1 (a hub node in phosphatidylinositol 3-kinase (PI3K)/Akt signaling), INS (insulin, regulating neuronal glucose metabolism), and IL-6 (a pro-inflammatory cytokine) emerged as the top three prioritized targets based on topological centrality, suggesting XYC may exert anti-epileptic effects by synergistically modulating neuroinflammatory cascades, metabolic homeostasis, and survival signaling pathways.

The PI3K/Akt signaling pathway, a central regulator of cellular survival and proliferation, is ubiquitously expressed in the central nervous system. In experimental models of epilepsy, activation of this pathway has been shown to modulate neuronal apoptosis and mitigate seizure severity, suggesting its dual role in neuroprotection and seizure suppression (66, 67). And AKT1 directly phosphorylates voltage-gated sodium channel NaV1.1, attenuating peak sodium currents (68), a pivotal mechanism regulating GABAergic neuronal excitability and ictogenesis.

Proinflammatory cytokines (PIC), including IL-1β, IL-6, and TNF-*α*, play pivotal roles in neuroinflammation and epileptogenesis by exacerbating neuronal hyperexcitability. Elevated PIC levels are consistently observed during epileptic seizures, with IL-6 demonstrating persistent upregulation across diverse epilepsy subtypes- even during interictal and postictal phases (69). Clinically, heightened serum IL-6 concentrations correlate with disease progression and may serve as a prognostic biomarker for epileptogenesis (70, 71).

Insulin functions as a critical neurotrophic factor, regulating synaptic plasticity, neurogenesis, and metabolic homeostasis within the central nervous system. Mounting evidence implicates cerebral metabolic syndrome—particularly insulin resistance—in the pathogenesis of neurodegenerative disorders and acute neurological injuries, including cerebral ischemia and epilepsy (72). And recent evidence suggests pediatric populations with obesity-driven insulin resistance exhibit a increased risk of developing epilepsy compared to metabolically healthy cohorts (73). This bidirectional interplay between metabolism and epilepsy is further exemplified by the metabolic sequelae of antiepileptic drugs: long-term valproate therapy induces hyperinsulinemia and insulin resistance in both adults and children, concomitant with elevated adiposity and dysregulated adipocytokine profiles (74). Our results indicating that, XYC could alleviate seizures in epileptic rats by affecting the expression of inflammatory cytokines and insulin resistance in central nervous system.

Based on the above findings, we have preliminarily elucidated the role and mechanisms of XYC in ameliorating hepatic lipid metabolism in NAFLD mice. However, it is important to acknowledge the limitations of translating these results to clinical applications. For instance, although lithium-pilocarpine-induced acute epilepsy rats models are well-established to simulating human epileptic seizures, their inability to fully recapitulate human pathophysiology, combined with the absence of clinical data, limits the direct clinical translation of our findings. Additionally, to further elucidate the molecular mechanisms by which XYC ameliorates the epileptic state in rats, subsequent studies will employ hippocampus-specific gene knockout models for in-depth investigation.

## 5 Conclusion

In this study, a multi-omics integrative approach (16S rDNA sequencing, untargeted metabolomics, and network pharmacology) was employed to elucidate the anti-epileptic mechanisms of XYC in a rat model. Metabolomic profiling identified glycerophospholipid metabolism as the predominant pathway modulated by XYC, with significant elevation of phosphatidylcholine (PC) and phosphatidylethanolamine (PE) levels. Gut microbiota analysis revealed that epilepsy-induced dysbiosis was partially reversed by XYC treatment, particularly through enrichment of SCFA producing *Lactobacillus* and suppression of pro-inflammatory *Enterobacteriaceae*. Network pharmacology further prioritized three core targets—AKT1, INS, and IL-6 —exhibiting strong binding affinities to XYC’s active compounds. Crucially, these targets functionally converge on glycerophospholipid homeostasis, mechanistically linking lipid remodeling (via AKT1/INS-mediated phospholipid biosynthesis) and neuroinflammation resolution (via IL-6 pathway inhibition) to XYC’s therapeutic efficacy.

## Data Availability

The original contributions presented in the study are included in the article/Supplementary material, further inquiries can be directed to the corresponding authors.

## Footnotes

^1^ http://rdp.cme.msu.edu/

^2^ http://bionet.ncpsb.org.cn/batman-tcm

^3^ https://www.omim.org/

^4^ https://www.disgenet.org/

^5^ https://www.genecards.org/
