# Supplementary material for: Xianyu capsule ameliorates neuroinflammatory and glycerophospholipid metabolism in lithium-pilocarpine-induced acute epilepsy
Source: Front Nutr. 2025 Aug 13;12:1625533. doi: 10.3389/fnut.2025.1625533 (PMC12381505; doi:10.3389/fnut.2025.1625533)
Supplement: Supplementary file 1 [file Table_1.DOCX]

# Supplementary materials

# Title: Xianyu Capsule ameliorates neuroinflammatory and glycerophospholipid metabolism in lithium-pilocarpine-induced acute epilepsy

**Authors:** Dongsheng Yu^1,#^, Shuang Li^2,#^, Xiaoping Li^1^, Xiaodan Zhang^3,4,*^, Danfeng Guo^3,4,*^

**Affiliations:**

^1^ Department of Chinese Medicine, The First Affiliated Hospital of Zhengzhou University, Zhengzhou, 450052, Henan, China

^2^ Department of Neurology, The First Affiliated Hospital of Zhengzhou University, Zhengzhou, 450052, Henan, China

^3^ Department of Hepatobiliary and Pancreatic Surgery, The First Affiliated Hospital of Zhengzhou University, Zhengzhou, 450052, Henan, China.

^4^ Henan Key Laboratory for Digestive Organ Transplantation, The First Affiliated Hospital of Zhengzhou University, Zhengzhou, 450052, Henan, China.

**Correspondence:** Prof. Danfeng Guo ([yfyguodf@zzu.edu.cn](mailto:yfyguodf@zzu.edu.cn)) and Dr. Xiaodan Zhang (zxdy2019@163.com)

^#^These authors contributed equally: Dongsheng Yu, Shuang Li.

**Running title**: XYC ameliorates epilepsy in rats

**Table S1 Relative content of metabolites in the Glycerophospholipids pathway (*n* = 6,** $\bar{\mathbf{x}}\boldsymbol{\pm SEM}$**)**

| No. | Name | Adduct | m/z | rt(s) | Control | Model | Model+XYC 0.7 |
| --- | --- | --- | --- | --- | --- | --- | --- |
| 1 | Glycerophosphate(2) | [M-H]- | 171.00 | 372.87 | 1.00±0.01 | 2.07±0.18^#^ | 1.05±0.01^*^ |
| 2 | Glycerophosphocholine | [M-H]- | 256.10 | 372.98 | 1.00±0.02 | 1.85±0.13^#^ | 1.03±0.02^*^ |
| 3 | sn-Glycerol 3-phosphoethanolamine | [M-H]- | 214.05 | 382.24 | 1.00±0.05 | 2.03±0.28^#^ | 1.18±0.08^*^ |
| 4 | Pi 36:5 | [M-H]- | 855.50 | 187.00 | 1.00±0.09 | 1.60±0.11^#^ | 1.24±0.07^*^ |
| 5 | 1-hexadecanoyl-2-sn-glycero-3-phosphate | [M-H]- | 409.23 | 245.01 | 1.00±0.08 | 1.75±0.20^#^ | 1.21±0.09^*^ |
| 6 | 1-stearoyl-2-hydroxy-sn-glycero-3-phosphate | [M-H]- | 437.26 | 242.66 | 1.00±0.08 | 1.63±0.13^#^ | 1.27±0.11^*^ |
| 7 | Pi 32:0 | [M-H]- | 809.51 | 189.14 | 1.00±0.21 | 2.53±0.40^#^ | 2.11±0.31 |
| 8 | Pi 34:2 | [M-H]- | 833.51 | 190.14 | 1.00±0.14 | 1.59±0.08^#^ | 1.42±0.08 |
| 9 | Pi 36:2 | [M-H]- | 861.54 | 189.45 | 1.00±0.15 | 1.55±0.05^#^ | 1.47±0.06 |
| 10 | Pc(16:0e/8-hepe) | [M+Hac-H]- | 840.57 | 136.26 | 1.00±0.05 | 0.84±0.06^#^ | 0.71±0.08 |
| 11 | 1-palmitoyl-2-hydroxy-sn-glycero-3-phospho-(1'-rac-glycerol) | [M-H]- | 483.27 | 163.21 | 1.00±0.10 | 1.55±0.23^#^ | 0.88±0.06^*^ |
| 12 | Pi(16:0e/15-hete) | [M-H]- | 859.53 | 103.89 | 1.00±0.16 | 1.79±0.32^#^ | 1.41±0.12 |
| 13 | 1-stearoyl-2-arachidonoyl-sn-glycero-3-phosphoserine | [M-H]- | 810.52 | 185.49 | 1.00±0.13 | 1.63±0.20^#^ | 1.42±0.14 |
| 14 | 1,2-dioleoyl-sn-glycero-3-phosphatidylcholine | [M+H]+ | 786.60 | 123.33 | 1.00±0.02 | 1.26±0.09^#^ | 0.99±0.04^*^ |
| 15 | 1-palmitoyl-2-linoleoyl-sn-glycero-3-phosphocholine | [M+H]+ | 758.56 | 296.99 | 1.00±0.05 | 1.95±0.27^#^ | 1.21±0.03^*^ |
| 16 | 1-palmitoyl-2-myristoyl-sn-glycero-3-phosphocholine | [M+Na]+ | 728.52 | 130.25 | 1.00±0.08 | 1.35±0.12^#^ | 1.14±0.06 |
| 17 | 1-hexadecanoyl-2-octadecadienoyl-sn-glycero-3-phosphocholine | [M+H]+ | 758.57 | 125.48 | 1.00±0.02 | 1.00±0.03 | 0.87±0.05^*^ |
| 18 | 1-o-hexadecyl-2-o-(2e-butenoyl)-sn-glyceryl-3-phosphocholine | [M+H]+ | 550.38 | 182.67 | 1.00±0.11 | 0.64±0.05^#^ | 0.74±0.04 |

^#^*P*<0.05 vs. the Control group; **P*<0.05 vs. the Model group.

**
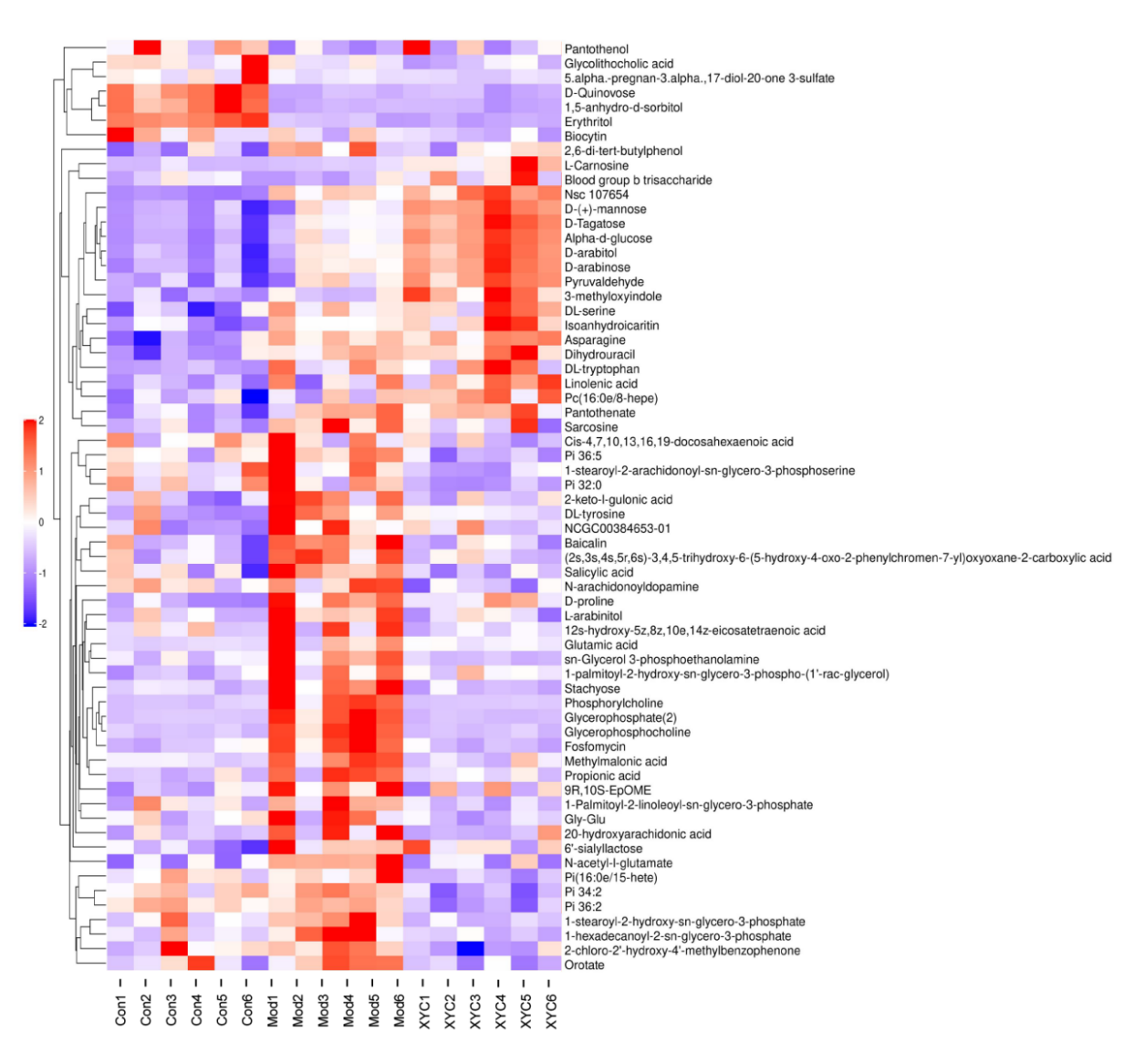
**

**Figure S1. The heatmap of altered serum metabolites of different groups in negative ion mode.**

**
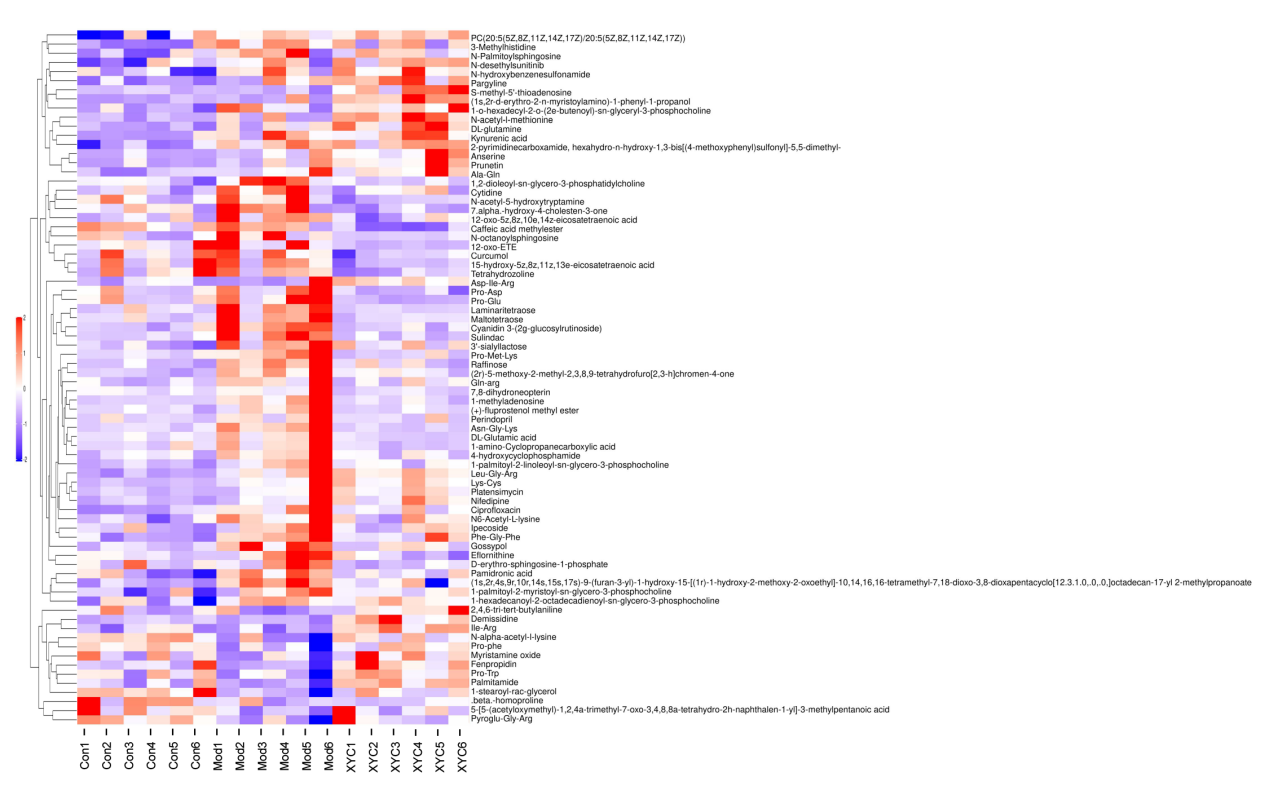
**

**Figure S2. The heatmap of altered serum metabolites of different groups in positive ion mode.**

**
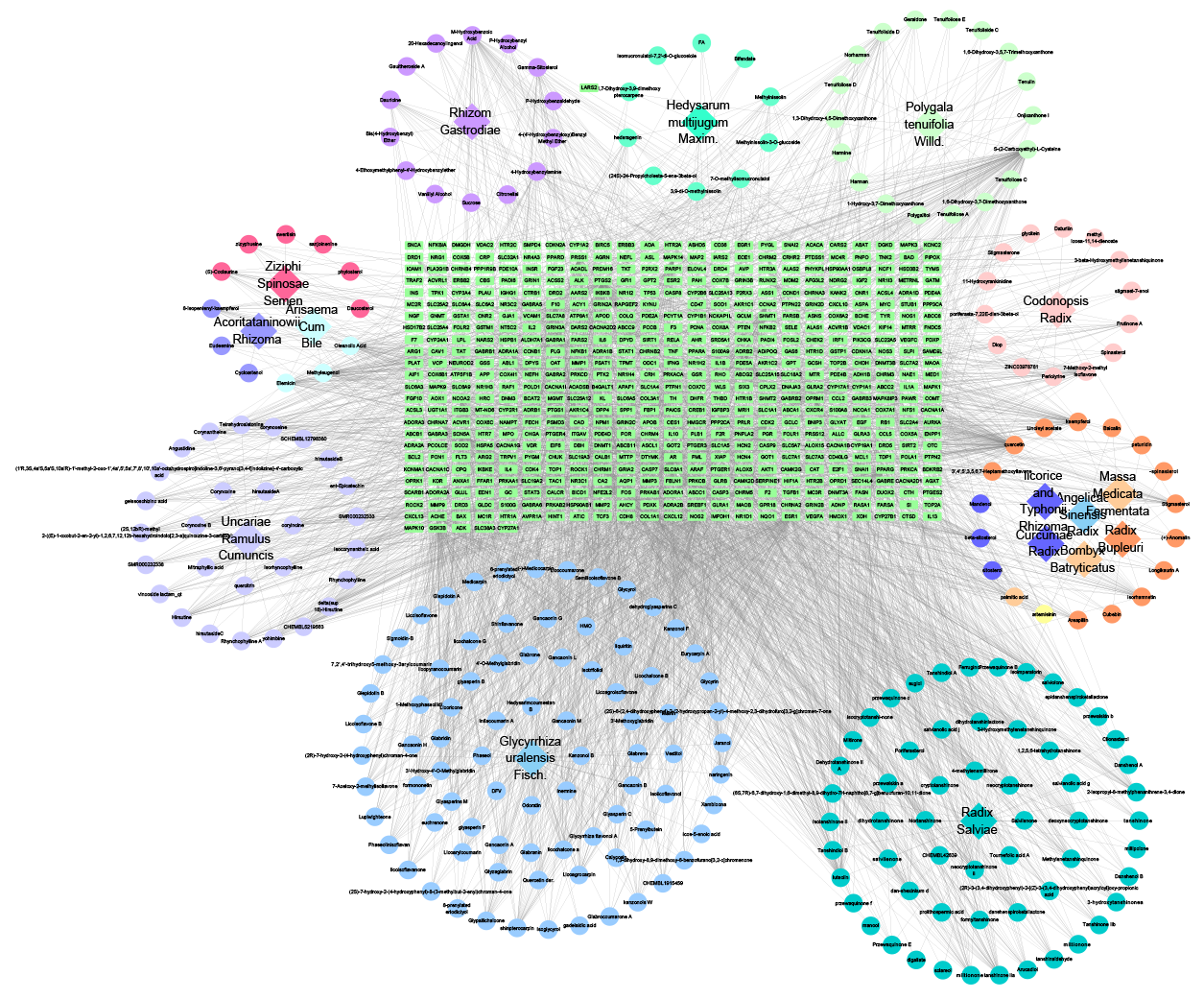
**

**Figure S3. The ingredient-target network of XYC in the treatment of epilepsy.**

**
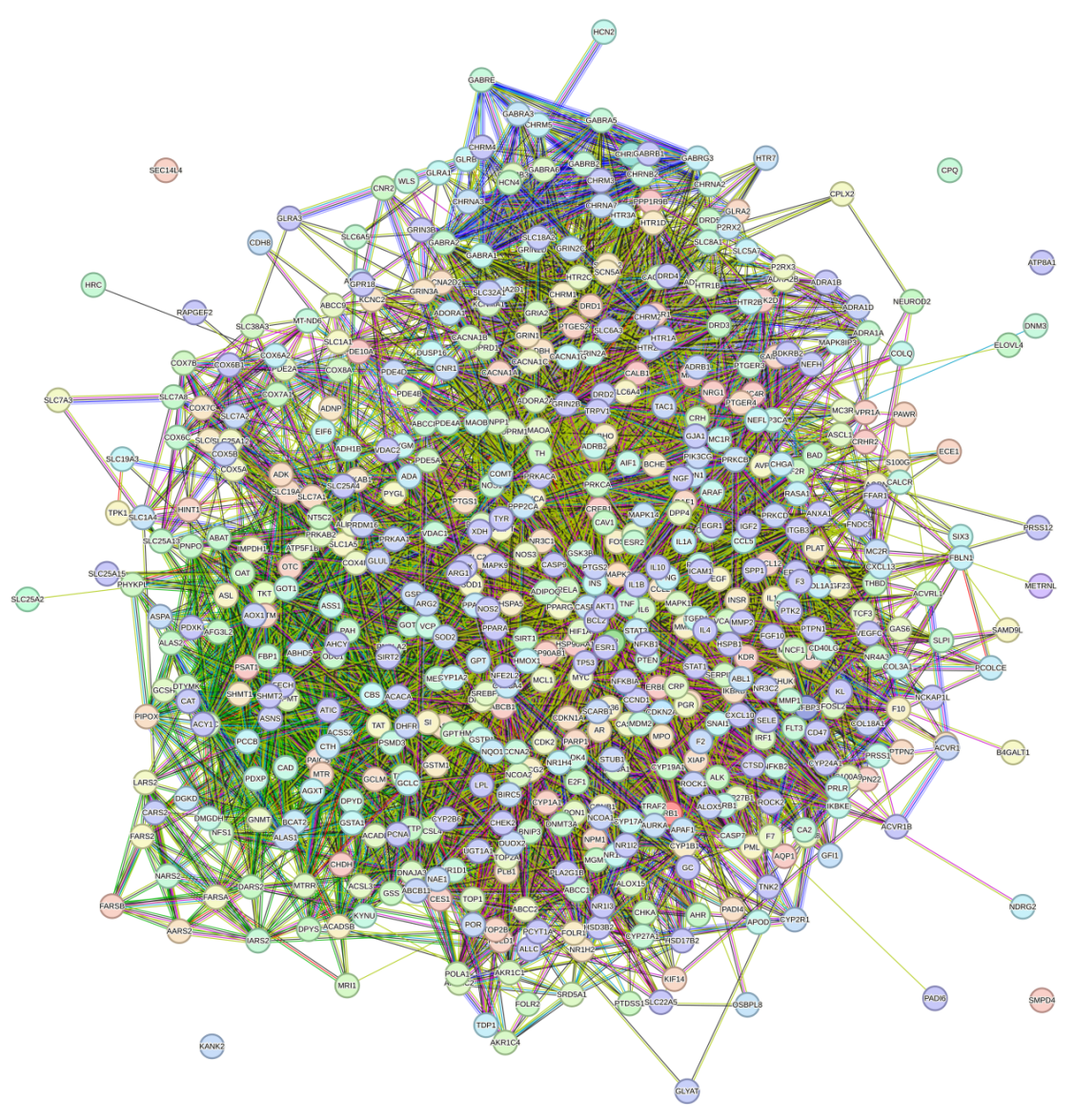
**

**Figure S4. The PPI network of XYC in treatment of epilepsy.**
